# Technical evaluation of methods for identifying chemotherapy-induced febrile neutropenia in healthcare claims databases

**DOI:** 10.1186/1472-6963-13-60

**Published:** 2013-02-13

**Authors:** Derek Weycker, Oleg Sofrygin, Kim Seefeld, Robert G Deeter, Jason Legg, John Edelsberg

**Affiliations:** 1 Policy Analysis Inc. (PAI), Brookline, MA, USA; 2 Amgen Inc, Thousand Oaks, CA, USA

**Keywords:** Febrile neutropenia, Neutropenia, Diagnosis, Classification, Sensitivity and specificity, Predictive value of tests

## Abstract

**Background:**

Healthcare claims databases have been used in several studies to characterize the risk and burden of chemotherapy-induced febrile neutropenia (FN) and effectiveness of colony-stimulating factors against FN. The accuracy of methods previously used to identify FN in such databases has not been formally evaluated.

**Methods:**

Data comprised linked electronic medical records from Geisinger Health System and healthcare claims data from Geisinger Health Plan. Subjects were classified into subgroups based on whether or not they were hospitalized for FN per the presumptive “gold standard” (ANC <1.0×10^9^/L, and body temperature ≥38.3°C or receipt of antibiotics) and claims-based definition (diagnosis codes for neutropenia, fever, and/or infection). Accuracy was evaluated principally based on positive predictive value (PPV) and sensitivity.

**Results:**

Among 357 study subjects, 82 (23%) met the gold standard for hospitalized FN. For the claims-based definition including diagnosis codes for neutropenia plus fever in any position (n=28), PPV was 100% and sensitivity was 34% (95% CI: 24–45). For the definition including neutropenia in the primary position (n=54), PPV was 87% (78–95) and sensitivity was 57% (46–68). For the definition including neutropenia in any position (n=71), PPV was 77% (68–87) and sensitivity was 67% (56–77).

**Conclusions:**

Patients hospitalized for chemotherapy-induced FN can be identified in healthcare claims databases--with an acceptable level of mis-classification--using diagnosis codes for neutropenia, or neutropenia plus fever.

## Background

Neutropenia is a common side effect of myelosuppressive chemotherapy. Neutropenia both increases the risk of infection and diminishes patients’ ability to fight infection. When neutropenic patients develop fever, a cardinal sign of infection, the high likelihood of serious consequences usually results in hospitalization for urgent evaluation, ongoing monitoring, and administration of intravenous antibiotics. This common and well-studied complication of neutropenia is called febrile neutropenia (FN), regardless of whether infection is ultimately documented as the cause of fever. FN--as well as severe or prolonged neutropenia--also has been shown to lead to chemotherapy dose-delays, dose-reductions, and discontinuation, interfering with the delivery of optimal treatment and possibly adversely affecting patient outcomes [1-4]. For these reasons, prophylactic administration of a colony-stimulating factor (CSF)--which has been shown to reduce the risk of FN, FN-related hospitalization, and infection, and to reduce antibiotic use in clinical trials--is recommended concurrently with myelosuppressive chemotherapy when FN risk is estimated to be approximately 20% or greater [3,5-8].

Using public and private healthcare claims databases as well as hospital discharge records, a number of retrospective studies have been undertaken to assess the clinical risk and economic burden of FN requiring hospitalization in clinical practice and to evaluate the comparative effectiveness of CSF agents against chemotherapy-induced hospitalized FN [7,9-14]. Because the study databases did not include information on absolute neutrophil count (ANC) and body temperature, and because there is no specific diagnosis code for FN, various combinations of codes for neutropenia, fever, and/or infection were utilized to identify FN in these studies. The accuracy of these definitions is unknown, however, and thus it is uncertain whether the methods of case ascertainment may have biased study results.

To the best of our knowledge, only one study has evaluated the test characteristics of claims-based definitions for FN, and this study included a small sample of older adults with a single tumor type (i.e., non-Hodgkin’s lymphoma) and evaluated only a single definition [15]. In light of the dearth of data on the accuracy of the claims-based definitions that have been used to identify FN hospitalizations, and because such data may be important in the design of future studies as well as in reducing bias in estimators based on definitions that are less than perfectly sensitive and specific (i.e., <100%), a new evaluation was undertaken [16].

## Methods

### Data source

Data were obtained from the MedMining Database, and spanned the period January 2004 through April 2010. The MedMining Database comprises electronic medical records (EMR) for services provided by the Geisinger Health System (GHS) to more than 2 million persons as well as healthcare claims for services received within GHS (as well as selected other health systems) to the approximately 225,000 members of the Geisinger Health Plan (GHP).

Data from the GHS EMR include: patient demographics (e.g., age, sex, race); ambulatory care visits and inpatient admissions (along with associated diagnoses [ICD-9-CM] and procedures [ICD-9-CM, HCPCS], and dates of service); medication orders/lists; and clinical laboratory and exam results (including white blood cell count, neutrophil count, body temperature, and dates of observation). Data from the GHP healthcare claims database include inpatient and outpatient diagnoses and procedures, outpatient drug utilization, and dates of service. EMR and healthcare claims data can be linked for all GHP members using unique patient identifiers, and all such data can be arrayed chronologically to provide a detailed longitudinal profile of medical and pharmacy services used within GHS by each GHP member.

Patient-identifying information was encrypted or removed from the study database prior to its release to the study investigators, as set forth in the corresponding Data Use Agreement. The study database has been evaluated and certified by an independent third party to be in compliance with the Health Insurance Portability and Accountability Act of 1996 (HIPAA) statistical de-identification standards and to satisfy the conditions set forth in Sections 164.514 (a)-(b)1ii of the HIPAA Privacy Rule regarding the determination and documentation of statistically de-identified data (45 CFR 46 §46.101). Use of the study database for health services research is therefore fully compliant with the HIPAA Privacy Rule and federal guidance on Public Welfare and the Protection of Human Subjects. Permission to use the data for this study was requested by study investigators and granted by MedMining.

### Source population

The source population included all GHP members, aged ≥18 years, who began one or more courses of myelosuppressive cancer chemotherapy between January 1, 2004 and April 30, 2010. Receipt of chemotherapy was ascertained based on the presence of one or more claims for a chemotherapy drug or administration, which were identified using HCPCS and ICD-9-CM codes in the healthcare claims database. Chemotherapy agents were classified by level of myelosuppression--any vs none--based on expert opinion. Regimens including one or more myelotoxic agents were considered to be myelosuppressive. Evidence of initiation of a new course of chemotherapy was based on the earliest claim for chemotherapy during the study period that was preceded by a 60-day or longer period without any other claims for chemotherapy; the date of the earliest such claim was designated as the “index date”. Among these identified patients, those with evidence of cancer as indicated by two or more medical claims with a qualifying 3-digit ICD-9-CM diagnosis code during the period beginning 60 days prior to the index date were selected for inclusion in the source population. For patients with multiple courses of chemotherapy (based on a gap of ≥60 days between the last administration of chemotherapy in a given course and the first administration in the next course) during the study period, all courses were considered. We excluded patients from the source population if they were not continuously eligible for comprehensive medical and drug benefits throughout the 60-day period prior to chemotherapy initiation. The classification of chemotherapy agents by level of myelosuppression is available in Additional file 1: Table S1 of the online supplement; cancer codes are available in Additional file 2: Table S2 of the online supplement.

### Chemotherapy cycles and regimens

For each patient in the source population, we identified each unique cycle within each course of chemotherapy. The first chemotherapy cycle was defined as beginning with the date of initiation of chemotherapy (i.e., the index date) and ending with the first service date for the next administration of chemotherapy (as evidenced by a medical claim with a corresponding HCPCS or ICD-9-CM code) occurring at least 7 days--but no more than 59 days--after the date of initiation of chemotherapy. If a second chemotherapy cycle did not commence prior to day 60, both the first cycle of chemotherapy and the course of chemotherapy were considered to have been completed 30 days following the beginning of the cycle. The second and all subsequent chemotherapy cycles were similarly defined, up to a maximum of nine cycles in total.

### Study population

The study population included all patients in the source population who were admitted to an acute care, short stay hospital in the GHS during the chemotherapy course--based on healthcare claims data--and who had an absolute neutrophil count (ANC) within 1 day of hospital admission (i.e., the day before, the day of, or the day after admission)--based on EMR data. Because chemotherapy-induced FN almost never occurs earlier than the fifth day of a chemotherapy cycle, and because some of the aforementioned retrospective studies did not consider hospitalizations during this period, only hospitalizations occurring on or after the fifth day of a chemotherapy cycle were included [11-13].

### Febrile neutropenia

The gold standard for identification of FN hospitalization (i.e., presumptive, for purposes of this retrospective evaluation) was evidence in EMR data of ANC <1.0 × 10^9^/L and either body temperature ≥38.3°C (101°F) or administration of antibiotic or antiviral therapy following ANC assessment, all having occurred within 1 day of hospitalization (i.e., the day before, day of, or day after hospitalization); this definition was considered the presumptive “gold standard” for purposes of analyses. Receipt of antibiotics was included in the definition because some patients (12%) had no recorded body temperature data during the period within one day of hospital admission, while others may have had fever prior to admission that had resolved with antipyretic therapy. We chose our gold standard definitions of neutropenia and fever to be as consistent as possible with those employed by the National Comprehensive Cancer Network (NCCN) and Infectious Diseases Society of America (IDSA), given the limitations of our database. The NCCN/IDSA defines neutropenia as ANC <0.5 × 10^9^/L or ANC <1.0 × 10^9^/L with a predicted decline to <0.5 × 10^9^/L over the next 48 hours. Because the study database does not include information on anticipated decline in ANC over time, and many patients--we believe--with ANC 0.5–1.0 would be expected to have ANC <0.5, we defined neutropenia as ANC <1.0 × 10^9^/L [17,18]. (We note that less authoritative guidelines have employed the higher threshold in their definition [19,20]). The NCCN/IDSA defines fever as a single temperature ≥38.3°C orally or ≥38.0°C over 1 hour. Because the study database does not provide information on duration of temperature readings, and because--we believe--the large majority of recorded readings were obtained orally, we defined fever as ≥38.3°C. In addition, because body temperature readings were not consistently recorded for all patients, our definition of fever was expanded to include administration of antibiotic or antiviral therapy, which was assumed to be a proxy for presence of infection and fever.

Claims-based operational definitions of FN hospitalization were based on ICD-9-CM inpatient diagnosis codes used in the aforementioned retrospective studies, and included: (1) neutropenia (ICD-9 288.0) in the primary position; (2) neutropenia in any position; (3) neutropenia plus fever (780.6) in any position; (4) neutropenia or fever in any position; and (5) neutropenia, fever, or infection in any position. (Because the last definition has been used in analyses to identify all neutropenic complications [including FN] resulting in hospitalization, its sensitivity was expected to be greater than the others, although at some loss of specificity and positive predictive value [PPV].) All of the aforementioned claims-based definitions were set forth on *a priori* basis, and thus were “blinded” to evaluation of the gold standard; however, operational definitions considering the ICD-9-CM diagnosis code for pancytopenia (284.1) (i.e., either neutropenia or pancytopenia) were evaluated on a “*post-hoc*” basis.

Each hospitalization was classified as either an FN hospitalization (FN positive) or a non-FN hospitalization (FN negative) based on each of the alternative claims-based definitions and gold standard, respectively. Thus, for each comparison of a claims-based definition versus the gold standard, four mutually-exclusive and mutually-exhaustive results were possible: (1) true-positive (FN positive by both definitions); (2) false-negative (FN negative by claims-based definition, FN positive by gold standard); (3) true-negative (FN negative by both definitions); and (4) false-positive (FN positive by claims-based definition, FN negative by gold standard) (Table 1).

**Table 1:** Definitions of test and performance characteristics

	**Gold standard**			
		Yes	No	
	Yes	X	Y	
	No	Z	K	
				
Test/Performance Characteristic		Definition		Explanation
Patient Classification, %				
True-Positive		X / (No. of Patients) × 100%		
False-Positive		Y / (No. of Patients) × 100%		
True-Negative		K / (No. of Patients) × 100%		
False-Negative		Z / (No. of Patients) × 100%		
Sensitivity, %		X / (X+Z) × 100%		% of FN hospitalizations correctly identified by claims-based definition
Specificity, %		K / (Y+K) × 100%		% of non-FN hospitalizations correctly identified by claims-based definition
Positive Predictive Value (PPV), %		X / (X+Y) × 100%		% of hosp. identified as FN hosp. by claims-based definition that were true-positives
Negative Predictive Value (NPV), %		K / (Z+K) × 100%		% of hosp. identified as non-FN hosp. by claims-based definition that were true-negatives
Accuracy, %		(X+K) / (No. of Patients) × 100%		% of all hospitalizations that were true-positives and true-negatives
Likelihood ratio positive (LR+)		[X/(X+Z)] / [1-K/(Y+K)]		Odds of hosp. being an FN hosp. when claims-based algorithm is positive
Likelihood ratio negative (LR-)		[1-X/(X+Z)] / [K/(Y+K)]		Odds of hosp. being a non-FN hosp. when claims-based algorithm is negative

FN: febrile neutropenia.

### Measures and analyses

The accuracy of claims-based definitions for FN, relative to the gold standard, was evaluated based on their test characteristics and performance characteristics, principally PPV and sensitivity; specificity, negative predictive value, accuracy, and positive and negative likelihood ratios also were evaluated. Hospitalizations misclassified by the claims-based definitions (i.e., false-positives and false-negatives) were further investigated and their diagnosis codes were reported. Confidence intervals for estimates of test and performance characteristics were calculated using techniques of nonparametric bootstrapping (percentile method) from the study population (1,000 replicates with replacement). Analyses were conducted using SAS® Software, Release 9.2 (SAS Institute Inc., Cary, NC).

## Results

A total of 3,193 patients received one or more courses of myelosuppressive chemotherapy during the period of interest, were continuously eligible for comprehensive medical and drug benefits during the 60 days prior to initiation of chemotherapy, and had evidence of cancer (Table 2). Among these patients, 772 (24%) had a hospitalization during a course of chemotherapy on or after the fifth day of a new cycle, and of these patients, 357 (46%) were hospitalized within GHS and had available data on ANC within 1 day of hospitalization, and thus were included in the study population. No patient in the study population had more than one qualifying hospital admission during the study period.

**Table 2:** Selection of source and study populations

**Inclusion/exclusion criteria**	**n (%)**
Source Population	
GHP members aged ≥18 years who received myelosuppressive chemotherapy from 01/01/04 - 4/30/10	3823 (100.0)
plus continuous health benefits during 60-day pretreatment period	3660 (95.7)
plus ≥2 claims with primary cancer diagnosis beginning 60 days prior to chemotherapy initiation	3193 (83.5)
Study Population	
plus admission to acute care, short stay hospital during chemotherapy course	772 (20.2)
plus admitted to hospital within GHS	373 (9.8)
plus ANC data available within 1 day of hospital admission	357 (9.3)

GHP: Geisinger Health Plan; GHS: Geisinger Health System; ANC: absolute neutrophil count.

Mean age of the study population was 64 (SD: 13) years and 56% of patients were male (Table 3). Mean ANC at the time of hospital admission was, on an overall basis, 4.4 × 10^9^/L. Common types of cancer included breast (10%), lung (19%), colon/rectum (11%), pancreas (8%), and non-Hodgkin’s lymphoma (11%). Thirty-five percent of subjects had metastatic disease, and 68% received chemotherapy with ≥2 myelosuppressive agents. Hospitalizations occurred most often in cycle 1 (28%) and cycle 2 (17%). Among the 357 patients in the study population, 82 (23%) met the gold standard for hospitalized FN; 28 (34%) of the 82 cases had low ANC, fever, and receipt of antimicrobial therapy, 30 (37%) had low ANC and receipt of antimicrobial therapy only (5 of the 30 were missing temperature data), and 24 (29%) had low ANC and fever only (all within 1 day of hospitalization).

**Table 3:** Patient, cancer, and chemotherapy characteristics

	**All patients (n=357)**	**Gold standard (n=82)**	**FN Patients: neutropenia, primary position (n=54)**	**FN Patients: neutropenia, any position (n=71)**	**FN Patients: Neutropenia + Fever,Any Position (n=28)**	**FN Patients: neutropenia or fever, any position (n=78)**	**FN Patients: neutropenia, fever, or infection, any position (n=201)**
Patient							
Age (years), mean±SD	64.2±13.0	62.1±13.8	63.3±15.1	62.6±14.8	61.3±16.3	63.0±14.5	65.5±12.3
Male, %	56.0	51.2	42.6	40.9	39.3	44.9	56.2
Body Mass Index (kg/m^2^), mean±SD	27.3±5.9	27.8±4.5	27.8±4.7	28.1±4.7	28.1±5.7	28.2±5.1	27.3±5.2
N	332	77	51	67	28	74	192
Absolute Neutrophil Count (cells/L), mean±SD	4.4±5.6	0.2±0.3	0.3±0.9	0.9±3.3	0.2±0.2	1.1±3.5	4.4±6.3
N	357	82	54	71	28	78	201
Cancer							
Type %							
Bone, Connective Tissue, Skin, Breast							
Female Breast	9.8	19.5	18.5	18.3	28.6	17.9	11.9
Other	2.8	1.2	1.9	2.8	3.6	2.6	2.5
Respiratory and Intrathoracic Organs							
Trachea, Bronchus, Lung	19.3	17.1	9.3	9.9	14.3	10.3	20.9
Other	1.4	2.4	5.6	4.2	3.6	3.8	2.0
Digestive Organs and Peritoneum							
Colon/Rectum	10.9	9.8	5.6	5.6	7.1	5.1	9.5
Pancreas	7.8	1.2	1.9	2.8	0.0	5.1	7.0
Esophagus	2.5	2.4	0.0	0.0	0.0	0.0	1.0
Other	3.1	0.0	0.0	0.0	0.0	0.0	2.0
Non-Hodgkin's Lymphoma	11.2	20.7	25.9	23.9	28.6	24.4	14.9
Genitourinary Organs							
Bladder	4.2	3.7	3.7	4.2	0.0	3.8	5.0
Ovarian	3.6	1.2	5.6	4.2	0.0	3.8	3.0
Prostate	2.8	0.0	0.0	0.0	0.0	0.0	3.0
Uterus	1.7	0.0	0.0	1.4	0.0	1.3	0.5
Other	2.8	1.2	1.9	1.4	0.0	1.3	2.0
Lip, Oral Cavity, and Pharnyx	2.0	1.2	0.0	0.0	0.0	0.0	1.5
Blood Cancers	6.7	13.4	13.0	14.1	14.3	14.1	8.0
Hodgkin’s Lymphoma	1.7	1.2	1.9	1.4	0.0	1.3	0.5
Other	5.6	3.7	5.6	5.6	0.0	5.1	5.0
Presence of Metastases, %	35.0	24.4	22.2	21.1	17.9	20.5	26.4
Chemotherapy							
No. of Myelosuppressive Drugs in Cycle 1, %							
1	32.2	20.6	26.1	27.4	38.1	29.4	29.2
2	47.2	50.7	45.7	46.8	38.1	44.1	49.7
≥3	20.6	28.8	28.3	25.8	23.8	26.5	21.1
Year of Initiation, %							
2004-2005	41.7	37.8	42.6	42.3	14.3	39.7	37.3
2006-2007	28.3	28.1	22.2	23.9	32.1	23.1	31.4
2008-2010	30.0	34.2	35.2	33.8	53.6	37.2	31.3

PPV was higher than 80% for claims-based definitions including the diagnosis codes for neutropenia plus fever in any position (PPV, 100%; sensitivity, 34% [95% CI: 24–45]) and neutropenia in the primary position (PPV, 87% [78–95]; sensitivity, 57% [46–68]) (Table 4). PPV was 77% (68–87) for the claims-based definition including the diagnosis code for neutropenia in any position; sensitivity was 67% (56–77). Sensitivity was highest 87% (79–94) when identifying hospitalized FN based on a diagnosis code for neutropenia, fever, or infection; PPV was 35% (28–42). The addition of the code for pancytopenia to the alternative claims-based definitions had minimal impact (i.e., it changed PPV and sensitivity by ≤3%).

**Table 4:** Test and performance characteristics of claims–based definitions for febrile neutropenia

			**Gold standard:**													
			FN:	No FN:		FN:	No FN:		FN:	No FN:		FN:	No FN:		FN:	No FN:
	Claims–Based Definitions:	FN:	47	7	FN:	55	16	FN:	28	0	FN:	55	23	FN:	71	130
		No FN:	35	268	No FN:	27	259	No FN:	54	275	No FN:	27	252	No FN:	11	145
																
																
Test/Performance Characteristic*			Neutropenia, Primary Position			Neutropenia, Any Position			Neutropenia + Fever,Any Position			Neutropenia or Fever, Any Position			Neutropenia, Fever, or Infection, Any Position	
Patient Classification, %																
True–Positive			13% (10–17)			15% (11–19)			8% (5–11)			15% (11–19)			20% (16–24)	
False–Positive			2% (0–3)			4% (2–7)			0% (–––)			6% (4–9)			36% (31–42)	
True–Negative			75% (70–79)			73% (68–77)			77% (73–81)			71% (66–75)			41% (36–45)	
False–Negative			10% (7–13)			8% (5–10)			15% (11–18)			8% (5–10)			3% (1–5)	
Sensitivity, %			57% (46–68)			67% (56–77)			34% (24–45)			67% (56–77)			87% (79–94)	
Specificity, %			97% (96–99)			94% (91–97)			100% (–––)			92% (88–95)			53% (47–59)	
Positive Predictive Value (PPV), %			87% (78–95)			77% (68–87)			100% (–––)			71% (61–80)			35% (29–42)	
Negative Predictive Value (NPV), %			88% (85–92)			91% (87–94)			84% (80–87)			90% (87–94)			93% (89–97)	
Accuracy, %			88% (85–91)			88% (85–91)			85% (82–89)			86% (82–90)			61% (55–66)	
Likelihood ratio positive (LR+)			22.5 (12.3–68.5)			11.5 (7.6–20.4)			–––			8.0 (5.6–13.4)			1.8 (1.6–2.1)	
Likelihood ratio negative (LR–)			0.4 (0.3–0.6)			0.3 (0.2–0.5)			0.7 (0.6–0.8)			0.4 (0.3–0.5)			0.3 (0.1–0.4)	

FN: febrile neutropenia.

*Value (95% CI).

For the claims-based definition identifying FN hospitalization based on a diagnosis of neutropenia in any position, eight of the 27 false-negatives patients had a diagnosis of septic shock/severe sepsis/septicemia, two had a diagnosis of pancytopenia, five had diagnoses of other infections, and 12 had neither a diagnosis of infection nor fever. Among the 16 false-positives, six patients had neutropenia (ANC<1,000), but none received antimicrobials and three had no temperature data recorded in the relevant time period. In addition, two patients, of whom one was febrile, had an ANC > 1,000 but < 1,500, and one had fever and received antimicrobial therapy with an ANC of 1,617.

## Discussion

Because they are easily accessible and provide readily-available information on a large number of patients receiving care in real-world clinical practice, private and public healthcare claims have been used extensively to date in health-economic and outcomes research. Such databases, however, are not without limitations, since they are not designed to capture certain components of care (e.g., laboratory results) and thus may lack data on potentially clinical parameters, which oftentimes are important for case-ascertainment. Inaccuracies in case-ascertainment may confer unknown biases in the estimation of study results.

In this study, we evaluated the accuracy of operational definitions previously used to identify chemotherapy-induced hospitalization FN in healthcare claims databases. We found that PPV exceeded 80%--the assumed minimum acceptable threshold--for the definitions including the diagnosis code for neutropenia in the principal position (87%) and diagnosis codes for neutropenia plus fever (100%), and that PPV was close to the acceptable threshold for the definition including a diagnosis code for neutropenia in any position (77%). Among these definitions, sensitivity was highest for the last one (67%), although this figure is somewhat lower than that (81%) reported for a similar definition in the aforementioned evaluation of older adults with non-Hodgkin’s lymphoma [15]. While sensitivity was improved in our study by including codes for fever and infection (87%), PPV (35%) was lower. We note, however, that this claims-based definition has most often been employed in comparative effectiveness studies of CSF agents, in which the focus was not on FN per se but all neutropenic complications (including severe neutropenia, afebrile infections in neutropenic patients, as well as FN) resulting in hospitalization that may be prevented with prophylaxis. When modifying the gold standard (i.e., ANC was < 1.5 × 10^9^/L) to capture additional neutropenic complications--irrespective of whether the patient was febrile or received antimicrobials--PPV (45% [38–53]) improved, but sensitivity was lower (81% [74–88]).

Because no one definition was found to be associated with both high PPV and high sensitivity, the selection of a specific definition for identifying hospitalized FN should be guided by the potential impact of misclassification on study outcomes. For example, in studies evaluating the comparative effectiveness of CSFs in clinical practice, a premium should be placed on a high PPV and limiting the inclusion of false-positive cases. Since CSFs can only reduce the risk of infections associated with true neutropenia, a low PPV would result in a dilution of the estimated effectiveness of CSF prophylaxis and estimated relative differences in effectiveness among agents being studied. A high PPV alone may not be sufficient, however, since if sensitivity is low, analyses may not be adequately powered to evaluate study objectives. For other studies, however, such as those in which the outcome is the cost of FN hospitalization, some misclassification may be acceptable to the extent that there are offsetting effects (e.g., if sensitivity and PPV are comparable; and either costs of false-positives, false-negatives, and true-positives are similar or costs of false-negatives and false-positives differ in the opposite direction by the same degree). The same also may be true for studies characterizing the clinical risk (i.e., incidence) of FN, for which--given the limitations of the alternative definitions--the goal should be to select one that is adequately sensitive and/or yields a balance between the number of patients who are misclassified (i.e., as false-positives and false-negatives). It therefore is essential to consider the importance of the individual test and performance characteristics of a measure, relative to the objectives of the study, before selecting one definition for use, and we recommend considering several alternative definitions.

Our study has a number of limitations. First, we excluded from the study population all patients who were not hospitalized in a GHS facility (because ANC data were not available for these patients) and those hospitalized in a GHS facility who did not have ANC data proximal to hospital admission. Only 35% of patients in the source population who were hospitalized (including patients treated inside and outside GHS) had ANC data during the study time period and thus could be included in the study population. For these reasons, the number of patients in the study population was limited to those treated at a GHS facility and who had ANC data. To the extent that excluded patients actually had FN and differed from the study population in important respects, our results may not be reflective of the larger population. In addition, because patients who had ANC data proximal to hospital admission may be selectively different from those who did not (because of, for example, a history of FN), our study population may be “enriched” and subject to verification bias. Second, even for some patients included in the study population, critical data--especially body temperature--was missing and thus we used a gold standard based on ANC and body temperature or administration of antibiotic or antiviral therapy. Because we used an “imperfect” gold standard, however, and thus patients’ true status in terms of FN was unknown, the presence of errors in the classification of patients by the gold standard could result in biased estimates of PPV, sensitivity, and other markers. Third, since the data for the study come from a single health plan and coding practices may vary across plans and geographic regions of the country, the generalizability of our findings is uncertain. Fourth, the accuracy of claims-based definitions in identifying FN may vary across subgroups of patients (e.g., those defined on the basis of type of cancer or regimen), and thus caution should be employed when generalizing study results to specific populations; such subgroup analyses, however, were not possible given the relatively small size of the study population. Fifth, while acknowledging that culture data are less than perfectly sensitive in identifying infection, the lack of such information in the data extract precluded any evaluation of the presence of infection in true FN patients based on positive cultures. Finally, because chemotherapy-induced FN almost never occurs earlier than the fifth day of a chemotherapy cycle, and because some of the aforementioned prior studies did not consider hospitalizations during this period, only hospitalizations occurring on or after the fifth day of a chemotherapy cycle were included. Results were not sensitive to this methodological feature as <5% of all hospitalizations occurred during the first five days of a chemotherapy cycle.

## Conclusion

In summary, the results of our study suggest that patients hospitalized for chemotherapy-induced FN can be identified in healthcare claims databases--with an acceptable level of mis-classification--using the diagnosis code for neutropenia or diagnosis codes for neutropenia plus fever. Research investigating the test and performance characteristics of operational definitions of FN using alternative databases (especially those in which ANC, body temperature, and culture data are available) is needed to verify the findings of this study, as is research in tumor-specific subgroups.

## Competing interests

Declaration of Funding. Funding for this research was provided by Amgen Inc. to Policy Analysis Inc. (PAI).

Declaration of Financial/Other Relationships. Derek Weycker, Oleg Sofrygin, Kim Seefeld, and John Edelsberg are employed by PAI. Robert Deeter and Jason Legg are employed by Amgen Inc. and own Amgen stock.

## Authors’ contributions

Authorship was designated based on the guidelines promulgated by the International Committee of Medical Journal Editors (2006). All persons who meet criteria for authorship are listed as authors. All authors have read and approved the final version of the manuscript. The study sponsor reviewed the study research plan and study manuscript, and was provided an opportunity to submit (non-binding) comments on study-related materials to study investigators. Data management, processing, and analyses were conducted by PAI, and all final decisions on data analytics as well as the content and structure of written materials--including the study manuscript--were made by study investigators. All authors read and approved the final manuscript.

## Pre-publication history

The pre-publication history for this paper can be accessed here:

http://www.biomedcentral.com/1472-6963/13/60/prepub

## Supplementary Material

Additional file 1: Table S1 Chemotherapy agents. Click here for file

Additional file 2: Table S2 ICD-9-CM codes for primary cancers. Click here for file
