# Supplementary material for: Technical evaluation of methods for identifying chemotherapy-induced febrile neutropenia in healthcare claims databases
Source: BMC Health Serv Res. 2013 Feb 13;13:60. doi: 10.1186/1472-6963-13-60 (PMC3576237; doi:10.1186/1472-6963-13-60)
Supplement: Additional file 1: Table S1 — Chemotherapy agents. [file 1472-6963-13-60-S1.doc]

| **Table S1 (online supplement).** Chemotherapy agents | | | | | |
| --- | --- | --- | --- | --- | --- |
| Agent | Level of Myelo-suppression | Agent | Level of Myelo-suppression | Agent | Level of Myelo-suppression |
| Aldesleukin | None | Doxorubicin Hydrochloride Liposome | High | Nelarabine | High |
| Alemtuzumab | Low | Epirubicin Hydrochloride | High | Nilotinib Hydrochloride | Low |
| Altretamine | Low | Eribulin Mesylate | High | Ofatumumab | High |
| Arsenic Trioxide | None | Erlotinib | None | Oxaliplatin | High |
| Asparaginase | Low | Estramustine Phosphate Sodium | None | Paclitaxel | High |
| Asparaginase Erwinia Chrysanthemi | Low | Etoposide | High | Panitumumab | None |
| Azacitidine | High | Etoposide Phosphate | High | Pazopanib Hydrochloride | Low |
| Bendamustine Hydrochloride | High | Everolimus | None | Pegaspargase | None |
| Bevacizumab | None | Floxuridine | Low | Pemetrexed | High |
| Bexarotene | Low | Fludarabine Phosphate | High | Pentostatin | Low |
| Bleomycin Sulfate | Low | Fluorouracil | High | Plicamycin | High |
| Bortezomib | High | Fulvestrant | None | Porfimer Sodium | None |
| Busulfan | High | Gefitinib | None | Pralatrexate | Low |
| Cabazitaxel | High | Gemcitabine Hydrochloride | Low | Procarbazine Hydrochloride | High |
| Capecitabine | High | Gemtuzumab Ozogamicin | None | Rituximab | None |
| Carboplatin | High | Hydroxyurea | High | Romidepsin | Low |
| Carmustine | High | Ibritumomab Tiuxetan | High | Sorafenib Tosylate | Low |
| Cetuximab | None | Ibritumomab Tiuxetan/Sodium Acetate | High | Streptozocin | Low |
| Chlorambucil | High | Idarubicin Hydrochloride | High | Sunitinib Malate | None |
| Cisplatin | High | Ifosfamide | High | Temozolomide | High |
| Cladribine | High | Ifosfamide/Mesna | High | Temsirolimus | None |
| Clofarabine | High | Imatinib Mesylate | Low | Teniposide | High |
| Cyclophosphamide | High | Irinotecan Hydrochloride | High | Thalidomide | None |
| Cytarabine | High | Ixabepilone | High | Thioguanine | Low |
| Cytarabine Liposome | High | Lapatinib Ditosylate | Low | Thiotepa | High |
| Dacarbazine | High | Leucovorin | None | Topotecan Hydrochloride | High |
| Dactinomycin | None | Lomustine | High | Tositumomab | High |
| Dasatinib | Low | Mechlorethamine Hydrochloride | High | Trastuzumab | Low |
| Daunorubicin Citrate Liposome | High | Melphalan | High | Tretinoin | None |
| Daunorubicin Hydrochloride | High | Melphalan Hydrochloride | High | Triptorelin Pamoate | None |
| Decitabine | High | Mercaptopurine | High | Valrubicin | None |
| Degarelix Acetate | None | Mesna | None | Vinblastine Sulfate | Low |
| Denileukin Diftitox | None | Methotrexate | High | Vincristine Sulfate | Low |
| Docetaxel | High | Mitomycin | Low | Vinorelbine Tartrate | Low |
| Doxorubicin Hydrochloride | High | Mitoxantrone Hydrochloride | High | Vorinostat | Low |
