# Supplementary material for: Technical evaluation of methods for identifying chemotherapy-induced febrile neutropenia in healthcare claims databases
Source: BMC Health Serv Res. 2013 Feb 13;13:60. doi: 10.1186/1472-6963-13-60 (PMC3576237; doi:10.1186/1472-6963-13-60)
Supplement: Additional file 2: Table S2 — ICD-9-CM codes for primary cancers. [file 1472-6963-13-60-S2.docx]

| **Table S2 (online supplement).** ICD-9-CM codes for primary cancers | |
| --- | --- |
| **Code** | **Description** |
| 140 | Malignant neoplasm of of lip |
| 141 | Malignant neoplasm of of tongue |
| 142 | Malignant neoplasm of of major salivary glands |
| 143 | Malignant neoplasm of of gum |
| 144 | Malignant neoplasm of of floor of mouth |
| 145 | Malignant neoplasm of of other and unspecified parts of mouth |
| 146 | Malignant neoplasm of of oropharynx |
| 147 | Malignant neoplasm of of nasopharynx |
| 148 | Malignant neoplasm of hypopharynx |
| 149 | Malignant neoplasm of other and ill-defined sites within the lip, oral cavity, and pharynx |
| **140-149** | **Lip, Oral Cavity, and Pharnyx** |
| 150 | Malignant neoplasm of esophagus |
| 151 | Malignant neoplasm of stomach |
| 152 | Malignant neoplasm of small intestine, including duodenum |
| 155 | Malignant neoplasm of liver and intrahepatic bile ducts |
| 156 | Malignant neoplasm of gallbladder and extrahepatic bile ducts |
| 157 | Malignant neoplasm of pancreas |
| 158 | Malignant neoplasm of retroperitoneum and peritoneum |
| 159 | Malignant neoplasm of in the digestive organs and peritoneum |
| **150-152, 155-159** | **Other Digestive Organs and Peritoneum (excluding colon and rectum)** |
| 153 | Malignant neoplasm of colon |
| 154 | Malignant neoplasm of rectum, rectosigmoid junction, and anus |
| **153-154** | **Colon and Rectum** |
| 160 | Malignant neoplasm of nasal cavities, middle ear, and accessory sinuses |
| 161 | Malignant neoplasm of larynx |
| 163 | Malignant neoplasm of pleura |
| 164 | Malignant neoplasm of thymus, heart, and mediastinum |
| 165 | Malignant neoplasm of other and ill-defined sites within the respiratory system and intrathoracic organs |
| **160-161, 163-165** | **Other Respiratory and Intrathoracic Organs (excluding trachea, bronchus, lung)** |
| **162** | **Trachea, Bronchus, and Lung** |
| 170 | Malignant neoplasm of bone and articular cartilage |
| 171 | Malignant neoplasm of connective and other soft tissue |
| 172 | Malignant melanoma of skin |
| 173 | Other malignant neoplasm of skin |
| 175 | Malignant neoplasm of male breast |
| 176 | Kaposi's sarcoma |
| **170-173, 175-176** | **Other Bone, Connective Tissue, Skin, Breast (excluding female breast)** |
| **174** | **Female Breast** |
| 179 | Malignant neoplasm of uterus, part unspecified |
| 180 | Malignant neoplasm of cervix uteri |
| 181 | Malignant neoplasm of placenta |
| 182 | Malignant neoplasm of body of uterus |
| 183 | Malignant neoplasm of ovary and other uterine adnexa |
| 184 | Malignant neoplasm of other and unspecified female genital organs |
| 186 | Malignant neoplasm of testis |
| 187 | Malignant neoplasm of penis and other male genital organs |
| 188 | Malignant neoplasm of bladder |
| 189 | Malignant neoplasm of kidney and other and unspecified urinary organs |
| **179-184, 186-189** | **Other Genitourinary Organs (excluding prostate)** |
| **185** | **Prostate** |
| 190 | Malignant neoplasm of eye |
| 191 | Malignant neoplasm of brain |
| 192 | Malignant neoplasm of other and unspecified parts of the nervous system |
| 193 | Malignant neoplasm of thyroid gland |
| 194 | Malignant neoplasm of other endocrine glands and related structures |
| 195 | Other and ill-defined sites |
| **190-195** | **Miscellaneous Other Sites** |
| 200 | Lymphosarcoma and reticulosarcoma |
| 202 | Other malignant neoplasms of lymphoid and histiocytic tissue |
| **200, 202** | **NHL** |
| **201** | **Hodgkin's Disease** |
